# Anti-Xa activities following 5,000 IU and 7,500 IU of s.c. dalteparin in critically ill patients and 5,000 IU in medical patients: a prospective randomized study

**DOI:** 10.1186/cc12289

**Published:** 2013-03-19

**Authors:** GH Heinz, U Priglinger, I Pabinger

**Affiliations:** 1 University Clinic of Internal Medicine II, Vienna, Austria; 2 University Clinic of Emergency Medicine, Vienna, Austria; 3 University Clinic of Internal Medicine I, Vienna, Austria

## Introduction

Unfractionated heparin is preferred over LMWH in ICU patients but LMWH is used more frequently in many European ICUs. Thromboprophylaxis with standard doses of nadroparin and enoxaparin has been shown to result in significantly lower anti-Xa in ICU patients when compared with medical patients [1,2].

## Methods

ICU patients (SAPS 44 ± 16, MV, *n = *44; pressors *n = *32) received 7,500 IU (Group 1, *n = *25) or 5,000 IU dalteparin s.c. (Group 2, *n = *29). Twenty-nine medical patients receiving 5,000 IU dalteparin served as controls (Group 3).

## Results

Group 2 had significantly lower areas under the Xa curve (AUC) compared with Groups 1 and 3 (Table 1). Differences were not significant between Groups 1 and 3. Peak anti-Xa activities (C_max_-anti-Xa) were delayed (t_max_-anti-Xa) in Group 2 compared with Groups 1 and 3 (Table 1).

**Table 1:** Pharmacokinetics

	Group 1 (*n *= 25) ICU, 7,500 IU	Group 2 (*n *= 29) ICU, 5,000 IU	Group 3 (*n *= 29) Normal ward, 5,000 IU	*P *overall
AUC-anti-Xa_0-12hours _(U/l^*^hour)	2.51 (1.15 to 4.61)	1.27 (1.15 to 4.4)	2.58 (1.45 to 4.87)	<0.001^*^
AUC-anti-Xa_12_00 _(U/l^*^hour)	1.37 (0.58 to 13)	1.47 (0.65 to 6.3)	0.89 (0.35 to 3.88)	0.003^**^
C _max_-anti-Xa (U/l)	0.29 (0.10 to 0.52)	0.14 (0.1 to 0.43)	0.33 (0.14 to 0.65)	<0.001^***^
t _max_-anti-Xa (hours)	3 (3 to 12)	4.5 (1 to 12)	3 (3 to 6)	0.017§

C_max' _peak anti-Xa level; t _max' _time of anti-Xa peak. Medians and range. ^*^Group 1 versus 2, *P = *0.001; Group 1 versus 3, *P = *0.51. ^**^Group 1 versus 2, *P = *0.96; Group 1 versus 3, *P = *0.045; Group 2 versus 3, *P = *0.13. ^***^Group 1 versus 2, *P = *0.001; Group 1 versus 3, *P = *0.29. ^§^Group 1 versus 3, *P = *0.09; Group 1 versus 2, *P = *0.8; Group 2 versus 3, *P = *0.018.

## Conclusion

In ICU patients a s.c. dose of 5,000 IU dalteparin results in significantly lower Xa activities when compared with normal ward patients. A s.c. dose of 7,500 IU dalteparin in ICU patients resulted in kinetics and peak anti-Xa activities comparable with medical patients receiving 5,000 IU dalteparin.
